# Advanced Diagnosis of Glioma by Using Emerging Magnetic Resonance Sequences

**DOI:** 10.3389/fonc.2021.694498

**Published:** 2021-08-05

**Authors:** Ruo-Lun Wei, Xin-Ting Wei

**Affiliations:** Department of Neurosurgery, The First Affiliated Hospital of Zhengzhou University, Zhengzhou, China

**Keywords:** glioma, radiomics, preoperative grading, differential diagnosis, 7-T magnetic resonance imaging, response assessment in neuro-oncology (RANO), magnetic resonance image

## Abstract

Glioma, the most common primary brain tumor in adults, can be difficult to discern radiologically from other brain lesions, which affects surgical planning and follow-up treatment. Recent advances in MRI demonstrate that preoperative diagnosis of glioma has stepped into molecular and algorithm-assisted levels. Specifically, the histology-based glioma classification is composed of multiple different molecular subtypes with distinct behavior, prognosis, and response to therapy, and now each aspect can be assessed by corresponding emerging MR sequences like amide proton transfer-weighted MRI, inflow-based vascular-space-occupancy MRI, and radiomics algorithm. As a result of this novel progress, the clinical practice of glioma has been updated. Accurate diagnosis of glioma at the molecular level can be achieved ahead of the operation to formulate a thorough plan including surgery radical level, shortened length of stay, flexible follow-up plan, timely therapy response feedback, and eventually benefit patients individually.

## Introduction

With its heterogeneous histological and imaging features, gliomas may still be the most common primary brain tumors in adults. The prognosis of patients with gliomas is not better than that of patients with other cancers, even for glioma patients who undergo various therapies such as aggressive surgery, chemoradiotherapy, and antiangiogenic therapy. Gliomas frequently occur in brain lesions and can be difficult to discern radiologically from other brain lesions, which might influence surgical planning and the course of follow-up treatment. In recent years, the development of magnetic resonance imaging (MRI) has greatly improved the clinical treatment and management of glioma patients. Previously, clinicians could only acquire basic information of tumor mostly from contrast-enhanced T1-weighted MR sequences. However, the pathophysiological aspects of gliomas can now be directly visualized and investigated with the help of emerging functional MR sequences. Currently, MRI plays a role throughout the course of the clinical treatment cycle. In addition to allowing the identification of different lesions in the central nervous system, the therapy plan can be elaborated in light of increasingly exquisite neuro-oncological imaging. With preoperative grading and key onco-marker detection, the formulation of individual treatment plans could contribute to improving prognosis and shortening the hospital length of stay (LOS). The correct radiological assessment during follow-up is crucial not only for the follow-up of glioma recurrence and progression but also for accurate assessment of therapeutic responses.

This review presents the frontiers of MR sequences in clinical applications relevant to the oncological imaging of glioma. The correlations between the MR sequences and their clinical applications in a glioma oncology diagnosis are discussed. Finally, the application of ultra-high-field MRI to glioma oncology is discussed.

## Noninvasive Preoperative Grading

Noninvasive preoperative grading and differential diagnosis of gliomas are useful for neurosurgeons. To differentiate between non-enhancing and enhancing brain tumors, amide proton transfer (APT)-weighted (APTw) MRI can be used in presurgical radiological assessments (1). As an indirect indicator of the cellular mobile protein content, APTw imaging has been well-received for its chemical exchange saturation transfer (CEST) technique, which allows visualization of changes in amide protons in the peptide bonds of mobile proteins that carry the water necessary for MRI. Routine subjoined APTw sequences in preoperative radiological examinations could be used for preliminary differentiation between low-grade (LGGs) and high-grade (HGGs) gliomas (1). The introduction of intravoxel incoherent motion (IVIM) MRI alongside APTw improved the efficiency of differentiation between LGGs and HGGs, with an area under the curve of 0.986 (2). Another systematic review that included 353 patients to evaluate the diagnostic performance of APTw MRI in differentiating between LGGs and HGGs indicated that HGGs have significantly higher amide proton-transfer signal intensity than LGGs (3). The pooled sensitivity and specificity were 88% and 91%, respectively. The clinical utility of APTw MRI was thus considered reliable.

Another powerful and advanced MRI technique is MR perfusion-weighted imaging (PWI), which can be used to visualize the aggressiveness and malignancy of a glioma. PWI facilitates the identification of the proliferation of neogenesis vessels and tumor angiogenesis in gliomas (4). Tumoral vessels can lead to hemodynamic changes in the brain due to their pathological structure, which is revealed in color maps of cerebral blood volume (CBV) and vessel wall permeability by means of PWI. This efficiency can be quantified as the relative CBV (rCBV), which is the ratio of tumoral CBV to normal-appearing white matter CBV. In PWI, increased CBV often reflects HGGs. At their first presentation, the rCBV of primary solid and non-enhancing WHO grade II gliomas on PWI was significantly lower in LGGs than that in HGGs (5). By setting the rCBV threshold at a fixed value for differentiation, dynamic susceptibility contrast-enhanced (DSC) PWI-derived rCBV is available and reliable for distinguishing newly diagnosed non-enhancing LGGs from HGGs.

As the traditional assessment for CBV, vascular-space-occupancy (VASO) MRI is impractical because of its low signal-to-noise ratio (6). Inflow-based VASO (iVASO) is improved by only inverting the blood flowing into the slice, which could reduce the partial effects of cerebrospinal fluid volume. Activating the iVASO response within a certain time window could maximize the reflection of arterial/arteriolar CBV (rCBVa) changes (7). Combining rCBVa from iVASO MRI with the minimum apparent diffusion coefficient (mADC) from diffusion-weighted imaging (DWI) shows higher preoperative grading efficiency than any sequence alone (8).

For the last decade, dynamic contrast-enhanced (DCE)-MRI has been a well-established technique for preoperative grading of gliomas (9, 10). The previous glioma grading model, mainly based on the “hot-spot” logic of DCE-MRI, recorded the average value of several well-visualized structures as the “hot-spot,” which advanced mapping of tumor boundaries but was deficient in measuring its heterogeneity. To quantify spatial variation in the grayscale intensity and depict the latent imaging heterogeneity, the use of textural features in DCE-MRI has advanced preoperative glioma grading. Textural features obtained from DCE-MRI, calculated by an algorithm and screened by the model, showed good efficiency of discrimination between grade III and IV gliomas. This had been impossible in the prior “hot-spot” model because of excessive homogeneity (11, 12).

The degree of intratumoral susceptibility signal (ITSS) of susceptibility-weighted imaging (SWI) helps to visualize normal vascular brain structures and the vasculature inside the glioma (13). HGGs tend to have greater micro-hemorrhage volume and vigorous angiogenesis under 3-T conventional MRI. Using a 7-T MRI scanner has a significant advantage over using its precursor in terms of spatial resolution due to its higher signal-to-noise ratio. Moreover, local image variance (LIV) is a new complementary technique that uses 7-T MRI for the quantification of hypointense microvascular SWI structures. Using LIV-SWI for quantitative analyses in preoperative gliomas, a significantly higher value can be found in HGGs than in LGGs, making 7-T MRI practical for preoperative grading (14).

Both rCBV from PWI and ITSS from SWI are capable of grading glioma noninvasively. The rCBV achieves this through comparing the CBV of tumor with white matter, while ITSS does this through visualizing the glioma vasculature. While the study by Park et al. (15) indicates that the degree of ITSS shows a significant correlation with the value of rCBVmax in the same tumor segments and the diagnostic performance of SWI on glioma grading is comparable to that of PWI, recent research further illustrated that glioma pathological type correlated with SWI ITSS score and WHO grade correlated with rCBV ratio (16). The combination of rCBV values and ITSS scores to improve grading accuracy is recommended (17).

## Differential Diagnosis

Glioma mostly manifests with neurological dysfunction, which can also be associated with other neoplastic and nonneoplastic lesions such as brain inflammation, lymphoma, or brain metastasis. Certain lesions require nonoperative treatments, rendering it necessary to distinguish them from gliomas. In clinical practice, clinical symptoms and preoperative examination features of these conditions often overlap, making them indistinguishable. The lack of a clear diagnosis may lead to invasive procedures such as biopsy, surgery, or even radiotherapy that may not only be inappropriate for the primary disease treatment but also eventually aggravate a patient’s condition. Therefore, it is imperative for clinicians to assess alternative noninvasive differential diagnostic tools to ensure an accurate preoperative assessment.

### Differential Diagnosis of Inflammation *vs.* Glioma

In routine clinical practice, the differentiation of brain parenchyma inflammation from grade II glioma may present a dilemma for neurosurgeons. Both inflammation and glioma manifest on conventional MR sequences as lesions with a mass effect. On certain sequences, they share the same characteristics, such as hypointensity on T1-weighted imaging (T1WI), hyperintensity on T2-weighted imaging (T2WI), and no enhancement on postcontrast T1WI. Radiomics analysis, a rapidly growing method, refers to the conversion of radiological images to quantitative data using a feature extraction algorithm. The radiomics algorithm overcomes the difficulties associated with the resolution limits of the bare human eye, making it an emerging and significant approach in clinical radiological assessments. By extracting several radiomics features from routine 3-T MRI sequences and integrating them into an algorithm, a radiomics model has achieved good diagnostic efficacy for distinguishing inflammation and glioma (18).

### Differential Diagnosis of Primary Central Nervous System Lymphoma *vs.* Glioma

Primary central nervous system lymphoma (PCNSL) is another common brain lesion with an increase in the incidence rate in recent decades due to the rising number of immunosuppressed and immunocompetent patients. PCNSL and HGGs share structural overlaps on MRI, both of which illustrate contrast-enhancing lesions with peritumoral edema. Quantitative APTw imaging analysis indicates that significantly higher homogeneous APTw hyperintensity, APTw min, and magnetization transfer ratio (MTR) and lower APTw max, APTw max-min, and CEST total signal intensity values can be found in PCNSLs than in HGGs. APT imaging is designed to detect free proteins and peptides in tissue. High MTR value and low CEST total signal of PCNSL lesions are often associated with a higher nuclear-to-cytoplasmic ratio. APTw max-min parameter indicates the APTw signal heterogeneity within the lesion. Significantly lower APTw max-min in PCNSL than in HGGs is consistent with the histopathological features that PCNSLs are histologically relatively homogeneous (19).

The peritumoral edematous areas of PCNSLs show significantly lower APTw value than that in HGGs, but the edema MTR values showed no statistical difference between these two types of tumors (19).

### Differential Diagnosis of Brain Metastasis *vs.* Glioma

Identifying a glioma from brain metastasis is another clinical predicament due to the similar symptoms of these conditions. PWI, an MRI sequence that can characterize the peritumoral area, theoretically ensures a high diagnostic performance in differentiating glioma from brain metastasis. Several sequences from PWI can play a vital role in clinical efficiency, such as DSC, DCE, and arterial spin labeling imaging. DSC is the PWI technique that is most commonly preferred by clinicians. Researchers tend to use rCBV as a DSC parameter to distinguish gliomas from brain metastases, as gliomas tend to invade adjacent brain tissue, whereas brain metastases tend to extrude adjacent brain tissue (20). In DCE-MRI, a glioma has higher peritumoral rCBV values than those in brain metastasis, indicating that the rCBV sequence can be practically used for target identification (21).

**Figure 1:**
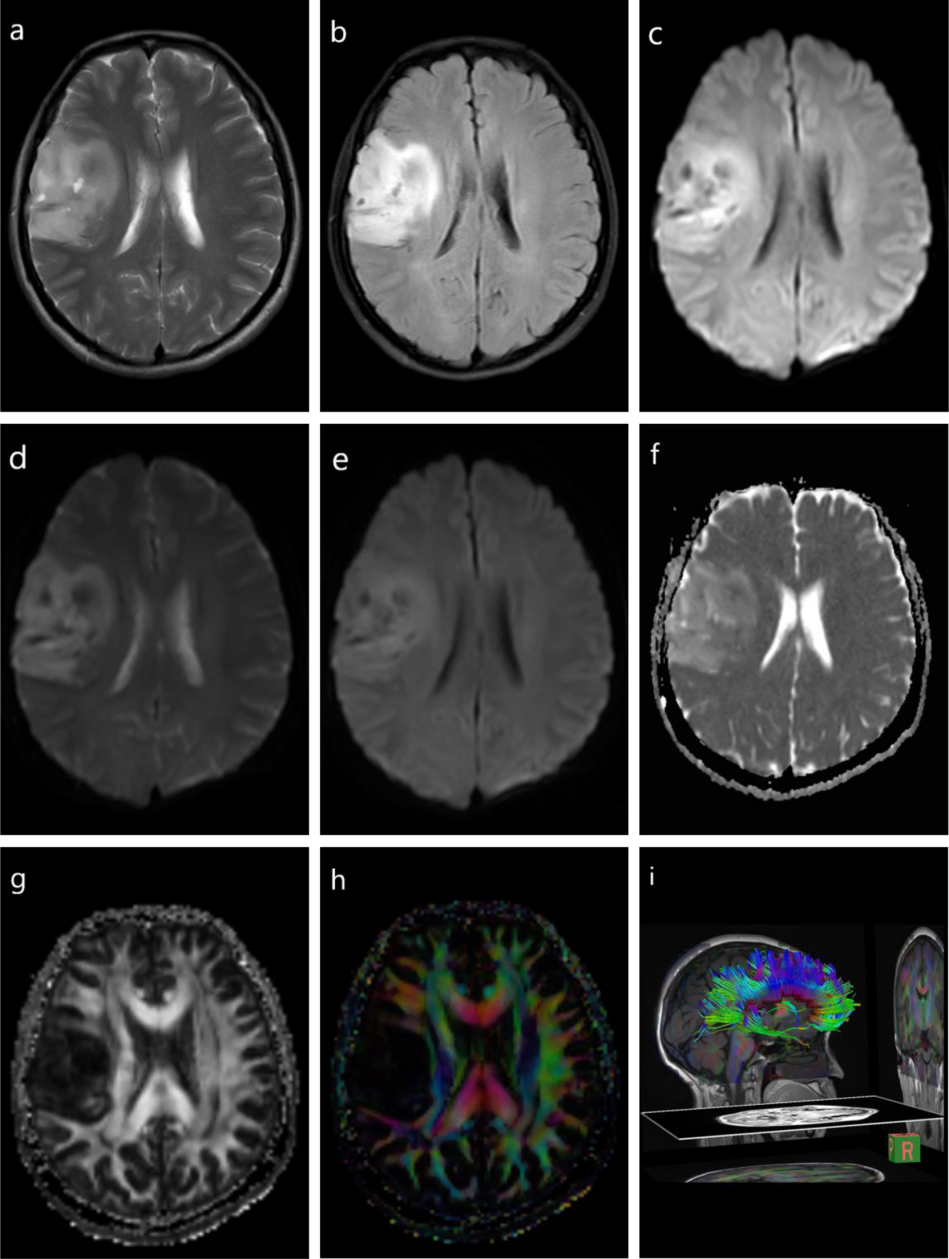
Diffusion-weighted imaging (DWI) and diffusion tensor imaging (DTI) of a glioma. T1-weighted imaging (T1WI) **(A)**, fluid-attenuated inversion recovery (FLAIR) **(B)**, DWI **(C)**, DWI with b = 0 s/mm^2^ **(D)**, DWI with b = 1,000 s/mm^2^ **(E)**, DTI apparent diffusion coefficient (ADC) mapping **(F)**, DTI fractional anisotropy (FA) mapping **(G)**, DTI directional encoded color FA mapping **(H)**, diffusion tensor tractography (DTT) **(I)**.

Numerous studies have reported distinguishing HGGs with solitary brain metastasis using DWI and diffusion tensor imaging (DTI) (22, 23). The focus of this differentiation is to distinguish infiltrative edema caused by the glioma from metastatic vasogenic edema. The mean minimum peri-enhancing ADC values in HGG are significantly higher than those in brain metastases, and combining DWI and DTI has shown a moderate diagnostic performance in this peritumoral area (24–26) (**Figure 1** and **Table 1**).

**Table 1:** MR sequence and parameter in glioma diagnosis.

Clinical application	Sequence	Parameter	Reference
**Preoperative grading**			
LGG *vs.* HGG	APTw	–	(1)
	PWI	rCBV	(5)
	iVASO+DWI	rCBVa+mADC	(8)
	7T-MRI	SWI-LIV	(14)
II *vs.* III (oligodendrogliomas)	DCE-MRI	Vp+Ktrans	(9)
III *vs.* IV	DCE-MRI	textural feature	(11, 12)
**Differential diagnosis**			
With PCNSL	APTw	–	(19)
With brain metastasis	PWI	rCBV	(20, 21)
	DWI+DTI	pADC	(22–26)
With inflammation	Radiomics (cMRI)	T1WI+T2WI	(18)
**Response to therapy**			
Identify tumor progression	DSC-PWI	rCBV	(27)
Response to TMZ	MRS	^1^H MRS	(28)
Response to standard CRT	CEST-MRI	MTR+NOE+APF	(29)
	7T CEST-MRI	rNOE	(30)

LGG, low-grade glioma; HGG, high-grade glioma; APTw, amide proton transfer weighted; PWI, perfusion-weighted imaging; rCBV, relative cerebral blood volume; iVASO, inflow-based vascular-space-occupancy; DWI, diffusion-weighted imaging; mADC, minimum apparent diffusion coefficient; SWI-LIV, susceptibility-weighted imaging local image variance; DCE-MRI, dynamic contrast-enhanced MRI; Vp, plasma volume; Ktrans, volume transfer coefficient; DTI, diffusion tensor imaging; pADC, peri-enhancing apparent diffusion coefficient; cMRI, conventional MR imaging; DSC-PWI, dynamic susceptibility-weighted contrast-enhanced perfusion-weighted imaging; MRS, MR spectroscopy; CEST-MRI, chemical exchange saturation transfer MRI; MTR, magnetization transfer ratio; NOE, nuclear Overhauser effect; APF, amide proton transfer; rNOE, relayed nuclear Overhauser effect.

## Key Onco-Marker Detection

### Isocitrate Dehydrogenase

Mutation of the metabolic enzyme isocitrate dehydrogenase (IDH) is one of the earliest known genetic events in the tumorigenesis of LGGs. Recent studies indicate that IDH may be a key driver in the development of multiple subtypes of LGGs. Molecular classification of gliomas has been revised due to the discovery of the IDH1 mutation. Gliomas with IDH1 mutations tend to have a more favorable prognosis.

Radiomics models can also play a vital role in identifying IDH-mutant gliomas ahead of surgery. After extracting the mADC, relative ADC (rADC), and rCBVmax from DWI, DSC-PWI, and conventional MRI data and integrating them into a prediction model, the mADC and rADC values were found to be higher in IDH-mutant gliomas than in IDH-wild-type gliomas. IDH-mutant gliomas also presented considerably lower rCBV values. The prediction model demonstrated a moderate diagnostic performance (31–33).

From a metabolic perspective, in IDH-mutant gliomas, isocitrate is not converted to α-ketoglutarate (α-KG) as usual but is converted to a new signature metabolite, 2-hydroxyglutarate (2-HG) (34). MR spectroscopy (MRS) is a noninvasive diagnostic modality that allows the detection and quantification of metabolites in cells and patients (35). Given that only IDH-mutant gliomas produce 2-HG, measuring 2-HG levels through MRS can estimate the IDH mutation status (36).

However, using MRS to identify IDH-mutant gliomas by detecting 2-HG has some drawbacks, as the process of correlating the 2-HG level and glioma tumor volume and MRS *per se* is time-consuming. Under APTw imaging, IDH-wild-type gliomas tend to demonstrate heterogeneous masses with scattered punctate or patchy high APTw signals, while IDH-mutant lesions show homogeneous iso-intensity to minimal APTw signals, making APTw a non-tumor volume-dependent and time-saving modality (37).

IDH1-R132H mutation status can also be predicted using 7-T CEST-MRI, with advanced diagnostic accuracy (p < 0.0001). The same MR sequence used in preoperative grading can be applied in IDH mutation status prediction, as IDH mutant gliomas manifest lower mean SWI-LIV values than IDH–wild-type gliomas (14).

### O6-Methylguanine-DNA Methyltransferase

Temozolomide (TMZ) is an oral alkylating agent that has been suggested to augment anti-glioma immune responses. Glioma patients may not respond to chemotherapy of alkylating agents due to the alkylator resistance caused by pivotal DNA repair enzyme such as O6-methylguanine-DNA methyltransferase (MGMT). Chemosensitivity to TMZ can be restored by methylating the MGMT promoter (MGMTpm). Therefore, MGMT promoter methylation is a robust indicator of glioma patients’ sensitivity to TMZ treatment. A radiomics approach using an automated machine-learning algorithm achieved moderate discriminatory accuracy of the MGMTpm status (38). Gd-3DT1WI, T2WI, and fluid-attenuated inversion recovery sequence (Flair) from 3-T MRI scans were integrated into the algorithm, of which the kernel is formed by applying the tree-based pipeline optimization tool (TPOT). The input features can thereafter be selected and classified, and the best machine-learning pipeline can be generated.

CBVa obtained from iVASO MRI can identify the difference of tumor histogram and structural features between MGMT methylation gliomas and unmethylation ones. The root mean square and variance features from CBVa histogram and contrast-enhancing component of the tumor location from structural imaging enable the iVASO-CBVa to evaluate the MGMT methylation status in gliomas (39).

### Histone H3-K27M Mutation

Compared to gliomas in other regions, diffuse midline gliomas (DMGs) mostly lead to a worse prognosis due to their diffuse growth pattern and high levels of intrinsic resistance to therapy. DMG occurs near the cerebral or infratentorial brain midline and occasionally intrudes into the spinal cord (40). DMG shares anatomical features with diffuse intrinsic pontine glioma (DIPG), a term that has now been abolished by the World Health Organization (WHO). In 2012, aberrations in a regulatory histone gene (H3) resulting in an amino acid substitution from lysine to methionine at residue 27 (K27M) were discovered in up to 40% of pediatric glioblastomas (41). Follow-up studies indicated that four out of every five childhood DIPG patients may possess H3-K27M mutations. These patients have a dismal prognosis (a mean of 0.73 years of survival), while those lacking the mutation survive for a mean of 4.6 years. Quantifying and qualifying the radiological features of the DWI in H3-K27M DMG, a moderately low ADC value compared to the H3-K27M wild type can be found in solid tumors (42). Through statistically different ADC values, several significantly lower parameters such as minimal ADC, peritumoral ADC, ratio of minimal ADC, and ratio of peritumoral ADC can be integrated to assess the H3-K27M mutational status in DMG (43).

### Ki-67

Proliferation-related Ki-67 is a representative antigen in the cell cycle. It has been widely used as a proliferation marker for human tumor cells. Ki-67 maintains low expression levels in normal brain tissues but is elevated in solid glioma tumors. More malignant tumors often possess a higher Ki-67 marker index and lead to worse prognosis. Therefore, it is imperative to assess the Ki-67 level preoperatively for better individual treatment (44). Fluctuations in texture features can be found in the peritumoral area of glioma due to the expression of Ki-67. As such, a radiomics model that integrates texture features from T1WI and T2WI can effectively assess the Ki-67 level noninvasively (45).

Conventional MRI can provide information about the volume, location, and texture of the tumor and inevitably suffers bias caused by the selected region of interest (46). Multicontrast MRI, the combination of multiple conventional MRI contrast sequences, could improve the objectivity of lesion detection (47). Multicontrast radiomics provides complementary information on both geometric characteristics and molecular biological traits, which correlate significantly with tumor proliferation. Under multicontrast MRI, non-wavelet and wavelet radiomics features were found to correlate significantly with the Ki-67 labeling index. The radiomics features and related parameters extracted from multicontrast demonstrated a good prediction of the Ki-67 level (48).

### P53

As a tumor suppressor gene, p53 has strong effects on gliomagenesis (49). p53 level often results in poor prognosis and malignant transformation of LGGs. Recent studies indicated that the sensitivity of gliomas to chemoradiotherapy may also be associated with the p53 level. Mutant p53 was found to be specifically associated with tumor location and enhancement texture maps in LGGs based on preoperative MRI scans (50). The least absolute shrinkage and selection operator (LASSO) method is an automated machine-learning approach that can select the best predictive features from the cohort to prevent the bias of overfitting and under-generalization caused by human selection. By integrating p53-related first-order (including maximum, median, minimum, and uniformity), shape- and size-based, and textural (including correlation, run percentage, and sum entropy) features, p53 level can be noninvasively and preoperatively predicted (51).

### Telomerase Reverse Transcriptase

The key for cancer cells to maintain their proliferative potential and avoid apoptosis is to maintain telomeres. Telomerase reverse transcriptase (TERT) is the rate-limiting catalytic subunit of telomerase. By increasing TERT expression, telomere length can be sustained (52). Mutations in the promoter region of TERT may facilitate TERT expression and serve as a crucial onco-marker in gliomas, particularly in glioblastomas (GBMs). Numerous studies have demonstrated that >80% of primary GBMs have a mutated TERT promoter (TERTpm) (53), indicating that it is fundamental to this tumor type. Volume of interest is a collection of key radiomics features selected by a specialized radiologist that can be further optimized to generate an optimal radiomics signature (Radscore). Based on a LASSO regression, multiple radiomics features, such as core necrotic volume percentages, Cho/Cr, Lac, and the Radscore, were found to be significantly higher in TERTpm than in TERT-wild-type tumors. Multiparameter models based on these statistically significant variables could predict the TERT promoter mutation status preoperatively (54).

### Alpha-Thalassemia/Mental Retardation, X-Linked

The function of alpha-thalassemia/mental retardation, X-linked (ATRX), as a chromatin remodeling protein is mainly expressed through histone variant H3.3. Distributed widely in gliomas, ATRX mutations contribute to the development of alternative lengthening of telomeres (ALT), and ATRX loss-of-function mutations have been confirmed to promote ALT (55). Based on the LASSO regression model, ATRX-associated radiomics features can be auto-selected. The ATRX status affects the overall image brightness, uniformity of the gray-level distribution, coarseness of an image, and symmetry of the image (56) (**Table 2**).

**Table 2:** Detection of onco-markers preoperatively by MRI.

Onco-markers	Sequence	Parameter	AUC	Reference
IDH mutation	cMRI+DWI+DSC-PWI	mADC+rADC+rCBVmax	0.92	(31)
	MRS	2-HG	–	(35)
	APTw	–	0.89	(36)
MGMTpm	Radiomics (TPOT)	3DT1WI+Gd-3DT1WI+T2WI+FLAIR	0.951	(37)
	iVASO	CBVa	0.931	(39)
H3-K27M mutation	DWI	ADC	0.872	(41, 42)
Ki-67 level	Radiomics (cMRI)	T1WI, T2WI	0.773	(43)
	Radiomics (multicontrast MRI)	ADC+eADC+CBF+PWmap+b1000+	0.745	(47)
		b0+T1FLAIR+T2FLAIR+T2FSE+T1C		
p53 mutation	Radiomics (cMRI)	T2WI	0.709	(50)
TERT	Radiomics (cMRI+MRS)	CE-T1WI+Flair+T1WI+T2WI	0.955	(53)
ATRX mutation	Radiomics (cMRI)	T2WI	0.94	(56)
EGFR amplified	Radiomics (cMRI)	T2WI	0.95	(57)
	DWI	mADC	0.75	(58)

IDH, isocitrate dehydrogenase; rADC, relative apparent diffusion coefficient; 2-HG, 2-hydroxyglutarate; MGMTpm, O6-methylguanine-DNA methyltransferase promoter methylation; TPOT, tree-based pipeline optimization tool; b1,000/b0 DWI with two b-values: b = 0 and b = 1,000 s/mm^2^; CE-T1WI, contrast-enhanced T1WI; TERT, telomerase reverse transcriptase; ATRX, alpha-thalassemia/mental retardation, X-linked; EGFR, epidermal growth factor receptor; mADC, minimum apparent diffusion coefficient.

### Epidermal Growth Factor Receptor

The epidermal growth factor receptor (EGFR) belongs to the ERBB family of tyrosine kinase receptors. EGFR signaling cascade is a key regulator in cell proliferation, differentiation, division, survival, and cancer development (59). EGFR overexpression can promote malignant proliferation of glioma cells, and several studies have focused on suppressing malignant proliferation by inhibiting its activity (60). A radiomics algorithm formed by texture features extracted from T2WI shows a good prediction of EGFR level in lower-grade gliomas. With 41 features validated and applied, the area under the curve (AUC) of the receiver operating characteristic (ROC) prediction curve reached the value of 0.95 (57).

Another study distinguished between glioma with amplified and non-amplified EGFR under DWI. EGFR-amplified tumor shows lower mean ADC values than EGFR-non-amplified gliomas, with an AUC of 0.75. Increased EGFR amplification has been associated with increased levels of cellular growth and proliferation. Higher EGFR amplification level reflects higher cellularity, which may lead to lower ADC values; thus, the mADC could independently predict the EGFR amplification level (58).

## Response to Therapy

In addition to overall survival, which is the gold standard of response to therapy, radiology-related measurements are increasingly favored by clinicians and radiologists. The Response Assessment in Neuro-Oncology (RANO) criteria were used for the accurate and reproducible assessment of responses to treatment in gliomas. The RANO criteria, which involve the radiology-based evaluation and measurement of tumors, can identify postsurgical progression in a timely manner, alter chemoradiotherapy plans, shorten clinical trial lengths, and reduce drug development costs (61).

### Identification of Tumor Progression

For most glioma patients, the glioma posttreatment radiation effect (PTRE) and tumor progression tend to occur in the first 2 years after surgery. Both present enhanced lesions, peritumoral irregular edema, space-occupying effects, and cystic necrosis, similar to conventional MRI- and CT-enhanced images. The treatment and prognosis of PTRE and tumor progression are quite different. PTRE often manifests as a positive response to adjuvant treatment and typically does not require further invasive treatment. Tumor progression, on the other hand, is a sign that the previous therapy had failed and should prompt treatment changes that may provide benefit. Therefore, it is crucial for clinicians to distinguish between PTRE and tumor progression to develop a proper treatment plan. Higher perfusion was observed in regions showing glioma progression due to active cell proliferation. In regions of PTRE, a lower perfusion status tends to be present because of capillary stenosis. This hemodynamic turmoil leads to rCBV variations. Due to the refined assessment possible with rCBV, DSC-PWI is a reliable tool for the timely identification of tumor progression (27).

### Temozolomide Responses

As an oral alkylating agent, TMZ is currently commonly administered to glioma patients due to it improving the adverse effects of traditional chemotherapy drugs. ^1^H MRS is a metabolic imaging method that can noninvasively measure several tumor metabolite levels. The levels of α-ketoglutarate and glutamate, intermediate products of mutant IDH gliomas, can be detected and quantitatively assessed by ^1^H MRS. In an *in vitro* experiment, after treatment with TMZ, the α-ketoglutarate and glutamate levels were found to be significantly lower than those in untreated glioma cells, indicating that ^1^H MRS may be a potential assessment tool for assessing the response to TMZ treatment in IDH1-mutant gliomas (28).

### Standard Chemoradiotherapy Responses

CEST is a recently emerged MR technique. Low concentrations of biomolecules can be detected using CEST by selective saturation of metabolite-bound protons and subsequent magnetization transfer to free water. This technology yields additional information about metabolic activity and the tissue microenvironment without the need for conventional contrast agents or radioactive tracers. CEST at 3-T reported good discrimination between glioma treatment responders and nonresponders. Using CEST-MRI to monitor the therapeutic response of gliomas to standard 6-week chemoradiotherapy (CRT) and several CEST matrices revealed significant differences, including in the MTR, nuclear Overhauser effect (NOE), and APT. Part of the matrix can even identify potential tumor progressors before the start of CRT (29).

The application of 7-T MRI allows the detection of more sophisticated and heterogeneous CEST effects. The relayed NOE signal in 7-T CEST-MRI scans allows direct distinction between responders and nonresponders immediately after the end of CRT. 7-T CEST-MRI enables early response assessments 4 weeks ahead of standard clinical evaluations, according to RANO (30).

## Clinical Feasibilities of Advance Sequences

APT technique was firstly invented in 2003 (62), and until now, it is the only noninvasive and non-radiative MR molecular imaging technology to be used for quantification of free protein. APT is currently applied for detecting brain tumors (63), grading gliomas, distinguishing active glioma from treatment effects, identifying genetic markers in gliomas, detecting ischemic stroke (62), and detecting Alzheimer’s disease (64) and Parkinson’s disease (65). Thus, APT technique is worth priority recommendation for hospitals with neurology and neurosurgery specializations.

Radiomics is an emerging field in quantitative imaging. Radiomics uses high-throughput extraction of advanced quantitative features to describe tumor phenotypes objectively and quantitatively. These features could be extracted from the existing medical images by advanced mathematical algorithms to uncover tumor characteristics that one may fail to appreciate by the naked eye. Radiomics features have shown promise in the prediction of treatment response (66), differentiating benign and malignant tumors (67), and assessing cancer genetics in many cancer types (68). With no extra hardware needed, radiomics can be quickly mastered and deployed to serve clinicians.

The most evident clinical application of 7-T is the higher spatial resolution in the brain compared to 3-T. In the last few years, studies indicate new insights into the pathology of the cerebral cortex on 7-T, such as cerebrovascular related neurodegenerative disease (69), multiple sclerosis (70), cortical microinfarcts (71), and mesial temporal lobe epilepsy (72). The higher spatial resolution contributes to the imaging of microvascular structures under SWI, which helps preoperative grading, and allows direct distinction between responders and nonresponders of CRT under 7-T CEST-MRI scans. For other aspects in glioma diagnosis, the clinical utility of 7-T MRI is yet to be explored.

For other techniques that require no upgrade of existing MR equipment such as rCBV from PWI and ADC from DWI, we recommend that neurosurgeons and radiologists utilize those sequences for glioma advance diagnosis at once.

## Conclusion and Future Directions

**Figure 2:**
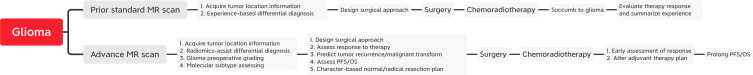
Glioma clinical practice flow under standard MR scan and advance MR scan.

Advanced MRI plays an increasingly important role in the clinical management of glioma by using emerging MR sequences to maximize safe resection, minimize surgery risk, individualize a CRT plan, shorten LOS, and ultimately prolong patients’ lives (**Figure 2**). A variety of parameters in multiple MR sequences are available for clinical use. The deployment of 7-T MRI with advanced morphological, functional, and metabolic imaging capabilities increasingly makes comprehensive diagnoses of gliomas possible. The correct and effective use of these MR techniques facilitates improved preoperative assessments for the accurate diagnoses and treatment responses of gliomas. Research focus on automatic machine learning and qualitative raw image data processing procedure that enables robust and thorough information for neurosurgeons will be crucial for enhancing glioma management in clinical routine. The correct and effective use of these MR techniques enables improved preoperative assessments of accurate diagnoses and treatment responses for glioma.

## Author Contributions

X-TW was responsible for the conception and design. R-LW wrote the manuscript. R-LW contributed to manuscript submission. All authors contributed to the article and approved the submitted version.

## Conflict of Interest

The authors declare that the research was conducted in the absence of any commercial or financial relationships that could be construed as a potential conflict of interest.

## Publisher’s Note

All claims expressed in this article are solely those of the authors and do not necessarily represent those of their affiliated organizations, or those of the publisher, the editors and the reviewers. Any product that may be evaluated in this article, or claim that may be made by its manufacturer, is not guaranteed or endorsed by the publisher.
